# Rita Levi-Montalcini: the neurologist who challenged fascism

**DOI:** 10.1055/s-0043-1761426

**Published:** 2023-03-14

**Authors:** Léo Coutinho, Hélio A. Ghizoni Teive

**Affiliations:** 1 Universidade Federal do Paraná, Hospital de Clínicas, Programa de Pós-Graduação em Medicina Interna, Curitiba PR, Brazil.

**Keywords:** History of Medicine, Neurology, Neurosciences, Nerve Growth Factor, Neurodegenerative Diseases, História da Medicina, Neurologia, Neurociências, Fator de Crescimento Neural, Doenças Neurodegenerativas

## Abstract

Rita Levi-Montalcini was a researcher in the field of neuroscience, Italian and Jewish in origin, who discovered the nerve growth factor and rightfully earned the 1986 Nobel Prize in Physiology or Medicine, alongside her collaborator Stanley Cohen. She was persecuted by the fascist dictatorship of Benito Mussolini and experienced gender and religious discrimination throughout her entire life. Despite these obstacles, she carried out her activities with diligence and grace, becoming a role model in the field. This paper reviews the life and career of Rita Levi-Montalcini.

## Resumo

Rita Levi-Montalcini foi uma pesquisadora no campo das neurociências, de origem Italiana e Judia, que descobriu o fator de crescimento neural e merecidamente recebeu o Prêmio Nobel de Fisiologia ou Medicina de 1986, em conjunto ao seu colaborador Stanley Cohen. Ela foi perseguida pela ditadura fascista de Benito Mussolini, e sofreu discriminação de gênero e religião durante sua vida inteira. A despeito desses obstáculos, sempre exerceu suas atividades com diligência e graça, tornando-se um exemplo nesse campo de estudo. O presente artigo faz uma revisão sobre a vida e carreira de Rita Levi-Montalcini.

## INTRODUCTION

Rita Levi-Montalcini (1909–2012) is among the most prestigious researchers in the history of neurobiology. For discovering the growth factors between 1952 and 1953, she received the 1986 Nobel Prize in Physiology or Medicine, alongside her collaborator, Stanley Cohen (1922–2020). 1 2 3 4 5 6 7 8 9

Early in her career, working in Italy during World War II, she had to overcome sanctions imposed by the fascist government of Benito Mussolini against “non-Aryan” academics. 1 2 3

This paper reviews the inspiring life and career of Professor Rita Levi-Montalcini, paying homage ten years after her death.

## A SHORT BIOGRAPHY

**Figure 1:**
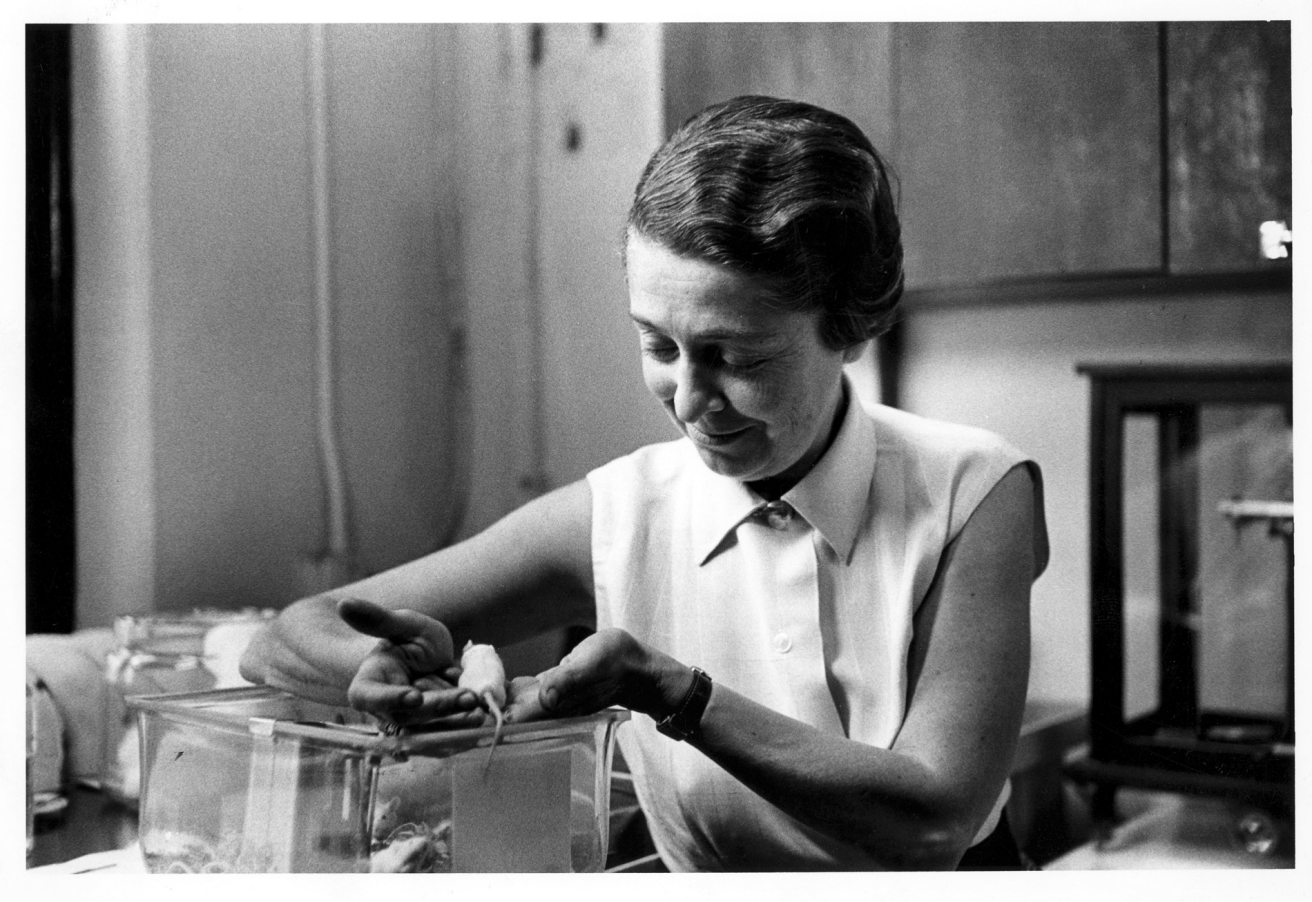
Rita Levi-Montalcini (1909–2012) in her laboratory, holding a mouse, circa 1959. Credit:Bernard Becker Medical Library Archives, Washington University School of Medicine.

Rita Levi-Montalcini ( Figure 1 ) and her twin sister Paola were born in 1909 in Turin, Italy. They were the youngest of four children to the Jewish couple Adamo Levi, an electrical engineer and mathematician, and Adele Montalcini, a painter. 1

Although her family was highly instructed, her father considered that a professional career would interfere with the traditional feminine role of wife and mother and decided that his daughters would not pursue higher education at a university. 1

Eventually, Rita realized she could not conform to this role, and at 20 years old, asked permission from her father to study and work. She entered medical school in Turin, where she graduated in 1936, followed by a three-year specialization in neurology and psychiatry. Following the promulgation of the *Manifesto per la difesa della razza* by the dictator Benito Mussolini, several laws were instituted impeding the academic and professional development of the “non-Aryan” population, jeopardizing Rita's incipient career. 1 2 3 4 5 6 7 8 9

In 1940, upon returning to Italy after a short period in Belgium, she assembled a research unit in her bedroom, starting her research influenced by a 1934 paper published by Viktor Hamburger on the effects of limb extirpation in chick embryos. 10 She worked in seclusion and under significant personal danger, with the sole collaboration of Giuseppe Levi, her former mentor at medical school. 1 2 3

She was forced to leave Turin in 1941 after the bombing by the allied forces, moving to the Piemonte, where she reconstructed her laboratory. In 1943 she was forced to leave her new facilities, moving to Florence, where she lived and worked underground until the end of the war; in Florence, she assisted the allied forces by working as a doctor in a refugee camp. 1 2 3

With the end of the war in Italy in 1945, she regained her position at the University in Turin, but in 1947 Professor Viktor Hamburger invited her to Washington University, in Saint Louis, to collaborate and repeat their experiments with chick embryos. Although she originally intended to remain there for one year, she remained until her retirement in 1977, after attaining the position of associate professor in 1956, and a full professorship in 1958. 1 2 3

During this period, she maintained close ties to Italy, establishing a research unit in Rome in 1962, and serving as director of the Institute of Cell Biology of the Italian National Council of Research from 1969 to 1978, becoming a guest professor after she retired from the institution. In 2002, she co-founded with Pietro Calissano the European Brain Research Institute, to which she was affiliated until she died in 2012, at 103 years old. 11

## THE DISCOVERY OF GROWTH FACTORS

Inspired by a 1948 article by Elmer Bueker, a student of Viktor Hamburger, reporting that chick embryos with implanted fragments of mouse sarcomas presented proliferation of the sensory nerve fibers into the neoplasm, 12 Rita Levi-Montalcini started to work to isolate the agent responsible for this proliferation. 13 14

However, her facilities lacked the structure necessary to perform her tissue cultures with adequate technique. She reached Professor Carlos Chagas Filho (1910–2000), and asked permission to carry out her research at the laboratory of Hertha Meyer (1902–1990) in the Biophysics department of Universidade Federal do Rio de Janeiro (formerly known as Universidade do Brasil). With the approval of Professor Carlos Chagas, she traveled to Rio de Janeiro in September 1952, carrying in her handbag two mice grafted with sarcomas, staying until January 1953. 1 2 3 9 13

She was accommodated in a house in Copacabana, which belonged to a friend of Hertha. Although she was fully dedicated to the research with sarcoma-bearing mice, she enjoyed herself in Rio. She went to the beach every day during the lunch hours, and sometimes entered the sea fully clothed, to change later in the Institute of Biophysics. Although – for her sadness – she departed shortly before the Carnival, she also engaged in the local festivities such as the celebrations of Iemanjá. 15

**Figure 2:**
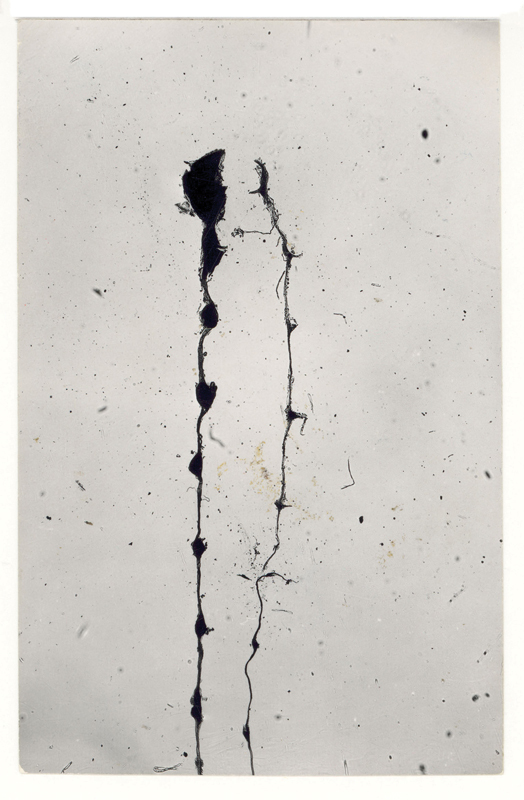
Nerve growth factor, circa 1959. Credit: Bernard Becker Medical Library Archives, Washington University School of Medicine.

After several negative results, she reached a turning point in her research: Her tissue cultures including sensory and sympathetic ganglia from chick embryos reacted to the sarcomas, promoting neurite growth and nerve cell differentiation. 8 Back in Washington, the young biochemist Stanley Cohen managed to isolate the substance responsible for this process of proliferation and differentiation: The nerve Growth Factor ( Figure 2 ). 1 2 3 13

At first, this discovery was received by the scientific community with suspicion and doubts about its relevance and applicability. Evidence towards the importance of the nerve growth factor, particularly in neurodegenerative diseases, piled on mainly after the 1970s, resulting in the researchers only receiving the due accolades with the Nobel in 1986. 7 16 17 18

## HER POLITICAL AND SOCIAL WORK

Throughout her life, Rita was very politically active. In 2001, she was made senator for life, and although she considered it a very stressful experience, she never lost a session. 11

She advocated for policies related to the valorization of science and education and having suffered from gender, race, and religious discrimination throughout her entire life, she condemned any form of prejudice. 1 2 3 11

She published the book “ *Le tue antenate* ” (Your ancestors), sharing the biographies and accomplishments of underrepresented women in science and social movements. She also created the Rita Levi-Montalcini Foundation to foster African girls to pursue a career in science, granting fellowships, particularly in medicine and nursing. 2 3 11

In conclusion, Rita Levi-Montalcini had a brilliant career, overcoming several obstacles, to become a role model for aspiring neuroscientists worldwide. Her discovery of the nerve growth factor paved the way for our current comprehension of neurodegeneration. 16 17 18
